# The Effect of *tonB* Gene on the Virulence of *Pseudomonas plecoglossicida* and the Immune Response of *Epinephelus coioides*

**DOI:** 10.3389/fmicb.2021.720967

**Published:** 2021-08-16

**Authors:** Lingfei Hu, Lingmin Zhao, Zhixia Zhuang, Xiaoru Wang, Qi Fu, Huabin Huang, Lili Lin, Lixing Huang, Yingxue Qin, Jiaonan Zhang, Qingpi Yan

**Affiliations:** ^1^Fisheries College, Jimei University, Xiamen, China; ^2^College of Environment and Public Health, Xiamen Huaxia University, Xiamen, China; ^3^Key Laboratory of Special Aquatic Feed for Fujian, Fujian Tianma Technology Company Limited, Fuzhou, China

**Keywords:** *Pseudomonas plecoglossicida*, *tonB*, pathogenicity, *Epinephelus coioides*, immune response

## Abstract

*Pseudomonas plecoglossicida* is the causative agent of “visceral white spot disease” in cultured fish and has resulted in serious economic losses. *tonB* gene plays a crucial role in the uptake of nutrients from the outer membranes in Gram-negative bacteria. The previous results of our lab showed that the expression of *tonB* gene of *P. plecoglossicida* was significantly upregulated in the spleens of infected *Epinephelus coioides*. To explore the effect of *tonB* gene on the virulence of *P. plecoglossicida* and the immune response of *E. coioides, tonB* gene of *P. plecoglossicida* was knocked down by RNAi; and the differences between the wild-type strain and the *tonB*-RNAi strain of *P. plecoglossicida* were investigated. The results showed that all of the four mutants of *P. plecoglossicida* exhibited significant decreases in mRNA of *tonB* gene, and the best knockdown efficiency was 94.0%; the survival rate of *E. coioides* infected with the *tonB*-RNAi strain was 20% higher than of the counterpart infected with the wild strain of *P. plecoglossicida*. Meanwhile, the *E. coioides* infected with the *tonB*-RNAi strain of *P. plecoglossicida* carried less pathogens in the spleen and less white spots on the surface of the spleen; compared with the wild-type strain, the motility, chemotaxis, adhesion, and biofilm formation of the *tonB*-RNAi strain were significantly attenuated; the transcriptome data of *E. coioides* infected with the *tonB*-RNAi strain were different from the counterpart infected with the wild strain of *P. plecoglossicida*; the antigen processing and presentation pathway and the complement and coagulation cascade pathway were the most enriched immune pathways. The results indicated that *tonB* was a virulence gene of *P. plecoglossicida*; *tonB* gene was involved in the regulation of motility, chemotaxis, adhesion, and biofilm formation; *tonB* gene affected the immune response of *E. coioides* to *P. plecoglossicida* infection.

## Introduction

*Pseudomonas plecoglossicida* is the causative agent of “visceral white spot disease” in *Epinephelus coioides* and *Larimichthys crocea* under the water temperatures of 15–20°C and has resulted in high mortality and heavy economic loss (Zhang et al., 2018; Huang et al., 2020). To alleviate the harm caused by *P. plecoglossicida*, its pathogenic mechanism has attracted much attention. The pathogenicity of pathogens was reported to be controlled by different genes. So far, many genes have been shown to have a strong relationship with the virulence regulation of aquatic pathogens, such as *sodA* and *sodB* to *Aeromonas hydrophila* (Zhang et al., 2019); *secA* and *cheB* to *Vibrio alginolyticus* (Guo et al., 2018); *ssaV* to *Edwardsiella piscicida* (Edrees et al., 2018); *toxA* and *toxB* to *Vibrio parahaemolyticus* (Singhapol and Tinrat, 2020); and *secY* and *tssD-1* to *P. plecoglossicida* (Luo et al., 2020; Ye et al., 2021). The previous transcriptome data (NCBI, SRP115064) of our lab showed that *tonB* gene of *P. plecoglossicida* was highly expressed in the spleen of *E. coioides*, which suggested that it might play a role in the virulence of *P. plecoglossicida*.

*tonB* gene encodes TonB, which is an element of the TonB system (TonB-ExbB-ExbD). The TonB system occupies a crucial position in the transport of nutrients, including iron, carbohydrates, hemin, transition metal elements, and vitamin B12 (Schauer et al., 2008; Huang and Wilks, 2017; Gomez-Santos et al., 2019). In Gram-negative bacteria, the TonB system and *tonB*-dependent transporter (TBDT) accomplish jointly transport of nutrients. TBDT can acquire energy to transport nutrients from the outer membrane with the help of the TonB system (Oeemig et al., 2018; Kopp and Postle, 2020; Samantarrai et al., 2020). Dong et al. (2019) found that the pathogenicity of *A. hydrophila* was attenuated due to the deletion of *tonB* gene. However, there have been no reports about *P. plecoglossicida tonB* gene.

Considering the great harms of *P. plecoglossicida* to aquaculture and the potentially important role of *tonB* in the pathogenicity of *P. plecoglossicida*, this article is devoted to exploring the contribution of *tonB* in the virulence of *P. plecoglossicida* and the immune response of *E. coioides* to *P. plecoglossicida* infection. *tonB* gene of *P. plecoglossicida* was stably knocked down by RNAi; the spleens at 3 and 5 day post injection (dpi) infected with the wild-type strain or the *tonB*-RNAi strain were sampled and subjected to RNA-seq to monitor the transcriptomes of *E. coioides*, and the transcriptome data were compared and analyzed.

## Materials and Methods

### Bacterial Strains and Culture Conditions

The virulent wild-type strain of *P. plecoglossicida* (NZBD9) was isolated from the spleen of large yellow croaker suffered from “visceral white spot disease” and was stored at −80°C (Huang et al., 2019). The *tonB*-RNAi strain of *P. plecoglossicida* was constructed from the wild-type strain of *P. plecoglossicida*. *P. plecoglossicida* was routinely grown in Luria Bertani (LB) broth under 18 or 28°C with shaking at 220 rpm. *Escherichia coli* DH5α was obtained from TransGen Biotech (Beijing, China) and grown in LB broth at 37°C with shaking at 220 rpm.

### RNAi-Induced Knockdown of *Pseudomonas plecoglossicida tonB* Gene

*Pseudomonas plecoglossicida tonB* gene was knocked down according to the methods described by Choi and Schweizer (2006) and Darsigny et al. (2010), with minor modifications. Four oligonucleotides complementary to short hairpin RNA (shRNA) sequences targeting *tonB* gene (Supplementary Table 1) were designed by BLOCK-iT^TM^RNAi Designer^1^ and synthesized by Shanghai Generay Biotech Co., Ltd. (Shanghai, China). The oligonucleotides were ligated to pCM130/tac vector using T4 DNA ligase (Takara Biomedical Technology, Beijing, China), according to the manufacturer’s recommendations. The recombinant plasmids were transformed into competent *E. coli* DH5α cells by heat shock and electroporated into *P. plecoglossicida* NZBD9 as described by Luo et al. (2019). The mRNA level of *tonB* gene in four mutants was determined by quantitative real-time polymerase chain reaction (qRT-PCR).

### Growth Rate Assay

The bacterial suspension were adjusted to optical density at a wavelength of 600 nm (OD_600_) = 0.3 and diluted 1,000-fold with LB broth. Aliquot of 200 μl of the bacterial diluent was added into per well of 96-well plate and incubated at 28°C. The OD_600_ values of bacterial culture were measured hourly for 48 h (Zuo et al., 2019). Ten replicates were carried out for each group.

### Bacterial Chemotaxis Assay

The bacterial chemotaxis assay for *P. plecoglossicida* was performed with fine-tuning as described by Zhang et al. (2020). The overnight culture of *P. plecoglossicida* was adjusted to OD_600_ nm ≈ 1.0 with sterile phosphate-buffered saline (PBS), and 0.25 ml of bacterial suspension was aspirated into a 1-ml syringe. Then, a capillary tube (inner diameter of 0.1 mm, one end sealed) filled with mucus was dipped into the bacterial suspension and incubated at 28°C for 1 h. Finally, the mucus in the capillary was blown out, and the colony-forming unit (cfu) number of *P. plecoglossicida* in the mucus was determined by dilution method of plate counting. Three replicates were performed.

### Biofilm Formation Assay

Biofilm formation assay of *P. plecoglossicida* was performed according to the method described by Mao et al. (2020), with some modifications. *P. plecoglossicida* at exponential growth period was adjusted to OD_600_ = 0.2 by fresh LB broth. Then, 100 μl of diluted bacterial suspension was added into per well of microtiter plate and incubated at 28°C for 24 h. After that, each well was washed twice with sterile PBS, dyed with 175 μl of crystal violet (0.1%) for 15 min, washed twice with sterile PBS, and air-dried. Finally, the stained biofilm was solubilized into 200 μl of 33% acetic acid, and the OD values of each well were measured at 590 nm. Eight independent replicates were performed.

### Motility Assay

The soft agar plate motility assay for *P. plecoglossicida* was performed as described by Zhang et al. (2019), with minor modifications. The overnight culture in LB was diluted with PBS and adjusted to OD_600_ = 0.3. Bacterial suspension of 1 μl was inoculated on LB semisolid agar plates supplemented with 0.4% agar and incubated at 28°C for 16–20 h. The diameters of bacterial colonies were measured. Biological replicates were carried out in triplicate for each group.

### Adhesion Assay

The bacterial adhesion assay was conducted according to the method depicted by Guo et al. (2018), with some modifications. Aliquot of 20 μl of sterile mucus was spread uniformly over the 22 × 22 mm glass slide area. After the mucus was air-dried, 4% methanol was used to fix mucus for 30 min. Bacterial suspension of 200 μl (OD_600_ = 0.3) was spread equably on the region of mucus on glass slides. Sterile PBS of 200 μl instead of bacterial suspension was used as the negative control. After incubation at 28°C for 2 h in a damp chamber, the slides were washed three times with PBS to remove the un-adhered bacterial cells. The adhering bacterial cells on the slide were fixed by 200 μl of 4% methanol for 30 min and dyed with 0.1% crystal violet for 3 min. After the unstained crystal violet were washed with PBS, the adhering bacterial cells in 10 randomly selected fields were counted under a microscope (×1,000). Five replicates were performed.

### *Epinephelus coioides* Infection and Sampling

All *E. coioides* infection experiments were executed completely following the proposals in the “Guide for the Care and Use of Laboratory Animals” set by the National Research Council (Copyright 1996 by the National Academy of Sciences). The animal protocols were officially ratified by the Animal Ethics Committee of Jimei University (Acceptance No. JMULAC201159).

Healthy weight-matched *E. coioides* were obtained from Zhangzhou (Fujian, China) and were acclimatized at 18 ± 1°C for 10 days in recirculating aquaculture systems.

For survival assay, size-matched *E. coioides* (60 fish per group) were intraperitoneally injected with the wild-type strain or *tonB*-RNAi strain of *P. plecoglossicida* at a dose of 5 × 10^4^ cfu/fish. In addition, 60 *E. coioides* intraperitoneally injected with PBS were used as the negative control. The daily mortality of experimental fish was observed and recorded until 10 dpi.

For RNA-seq, six spleens of three different groups (wild-type strain group, *tonB*-RNAi strain group, and PBS group) were sampled at 3 and 5 dpi, and two spleens were mixed into one sample. All of the samples were sent to Shanghai Majorbio Bio-pharm Technology Co., Ltd. (Shanghai, China) for sequencing.

For the pathogen load assay and *tonB* expression assay, six spleens of *E. coioides* intraperitoneally infected with the wild-type strain or *tonB*-RNAi strain of *P. plecoglossicida* were randomly sampled at 1, 2, 3, 4, 5, and 6 dpi. *P. plecoglossicida* cultured at 18°C *in vitro* was considered as the control.

### Quantitative Real-Time Polymerase Chain Reaction

Quantitative real-time polymerase chain reaction was performed by a QuantStudio 6 Flex real-time PCR system (Life Technologies, Carlsbad, CA, United States). Primers are synthesized by Xiamen Borui Biotechnology, and primer sequences are provided in Supplementary Table 2. The 16S rDNA (Sun et al., 2019) was applied to normalize *tonB* gene expression levels of *P. plecoglossicida*. The pathogen load of *P. plecoglossicida* in the infected spleens was assessed by the copy number of housekeeping gene *gyrB* (Xin et al., 2020). The relative expression of gene in different groups was calculated using 2^–Δ^ ^Δ^ ^*CT*^ method (Luo et al., 2020).

### Transcriptomic Analysis

A TruSeq^TM^ RNA sample preparation Kit (Illumina, San Diego, CA, United States) was used to prepare the RNA-seq libraries under protocols of the Kit. The RNA quality was detected and quantified by the Agilent 2100 Bioanalyzer system (Agilent Technologies, Santa Clara, CA, United States) and the ND-2000 instrument (NanoDrop Technologies, Thermo Fisher Scientific, Waltham, MA, United States) separately. The high-quality RNA samples [OD260/280 = 2.03–2.09, RNA integrity number (RIN) ≥ 9.0, 28S:18S ≥ 1.0, ≥1.6 μg) met the construction of individual sequencing libraries. The rRNA-depleted RNA samples were fragmented in fragmentation buffer, and cDNA synthesis was carried out with protocols supplied with the SuperScript double-stranded cDNA synthesis kit (Invitrogen, Carlsbad, CA, United States). The cDNA libraries were amplified by Phusion DNA polymerase (NEB) after end repair, phosphorylation, and poly(A) addition. Sequencing was performed on the Illumina HiSeq4000 sequencing platform at Majorbio Biotech Co., Ltd. (Shanghai, China). The trimming and quality control of the raw Illumina reads were performed using SeqPrep^2^ and Sickle^3^ with the default settings. The mapped reads were used for *de novo* assembly as the unigenes of *E. coioides*.

To investigate the biological processes, the BLAST2GO software^4^ was used for the Gene Ontology (GO) annotation (Conesa et al., 2005), which accomplished the molecular annotation of differentially expressed transcripts of *E. coioides*. The differentially expressed mRNAs (DEMs) met the standards [| log2FC| ≥ 1 and false discovery rate (FDR) < 0.05] was deemed as significant. The GO enrichment analysis visualization of *E. coioides* transcriptome data was performed by the clusterProfiler R package (Yu et al., 2012). Finally, metabolic pathways were analyzed with Kyoto Encyclopedia of Genes and Genomes (KEGG) (Okuda et al., 2008). Furthermore, 10 genes were randomly selected from *E. coioides* to verify the reliability of RNA-seq by qRT-PCR (Supplementary Figure 1).

### Statistical Analyses

The experimental data are showed as means ± SD and dissected with one-way ANOVA followed by Dunnett’s test using IBM SPSS Statistics 26.0 (Armonk, NY, United States). *p* < 0.05 was considered as statistically significant.

### Data Access

The RNA sequencing results were put aside in the GenBank SRA database under accession number SRP315640.

## Results

### Effect of RNAi on *tonB* mRNA Level of *Pseudomonas plecoglossicida*

**FIGURE 1:**
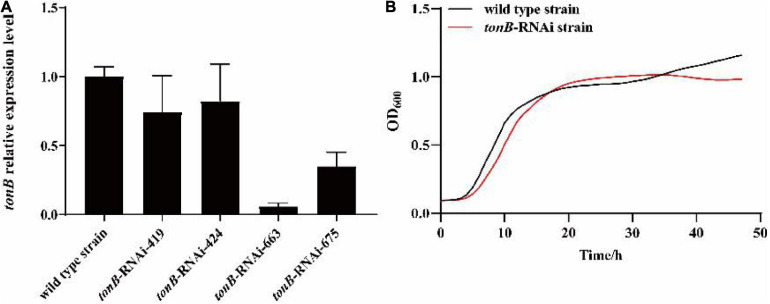
Construction and growth curve of the *tonB*-RNAi strain of *Pseudomonas plecoglossicida*. **(A)** The *tonB* mRNA levels of four mutant strains. **(B)** Growth curve of the wild-type strain and *tonB*-RNAi strain.

After shRNA sequence design and synthesis, recombinant plasmid construction, and electrical transfer, four RNAi mutant strains (*tonB*-RNAi-419, *tonB*-RNAi-424, *tonB*-RNAi-663, and *tonB*-RNAi-675) were successfully constructed. The results of qRT-PCR showed that *tonB* gene mRNA levels of four mutants were lower than those of the wild-type strain of *P. plecoglossicida* (Figure 1A). The mutant named *tonB*-RNAi-663 (hereafter called the *tonB*-RNAi strain) exhibited the best silencing efficiency (94.0%) and was chosen for further research.

### Effects of *tonB* Gene Silencing on the Growth of *Pseudomonas plecoglossicida*

The growth curve of the *tonB*-RNAi strain and wild-type strain of *P. plecoglossicida* under the same culture conditions was determined. The results illustrated that there was no significant difference between the growth rate of the *tonB*-RNAi strain and wild-type strain of *P. plecoglossicida* (Figure 1B).

### Effects of *tonB* Gene Silencing on the Characteristics of *Pseudomonas plecoglossicida*

**FIGURE 2:**
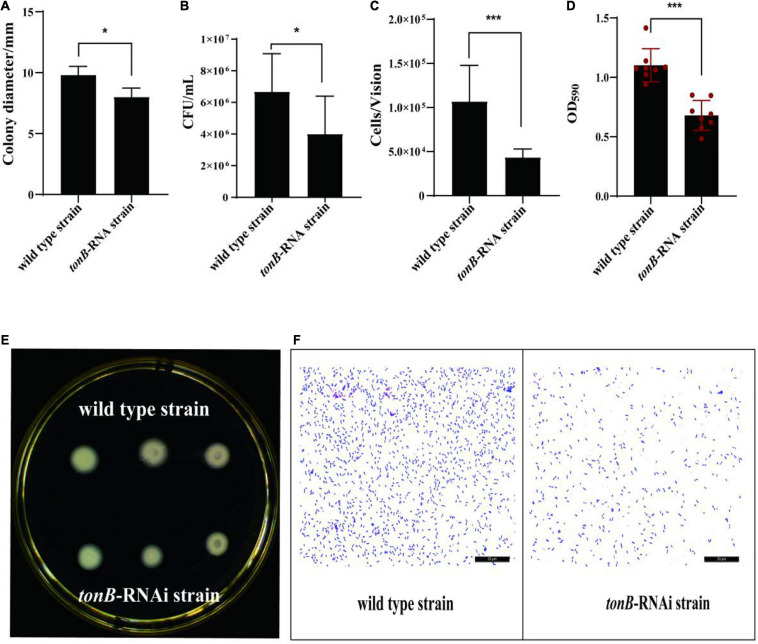
The characteristics of two strains of *Pseudomonas plecoglossicida*. **(A)** Colony diameters of wild-type strain and *tonB*-RNAi strain. **(B)** Chemotaxis capacity of wild-type strain and *tonB*-RNAi strain. **(C)** Adhesion ability of wild-type strain and *tonB*-RNAi strain. **(D)** Biofilm formation ability of wild-type strain and *tonB*-RNAi strain. **(E)** Colonies of wild-type strain and *tonB*-RNAi strain on a plate. **(F)** Adhering bacteria under the microscope. Data are presented as mean ± SD. *p < 0.05, **p < 0.01, ***p < 0.001.

The motility, chemotaxis, and adhesion of the *tonB*-RNAi strain of *P. plecoglossicida* were enervated compared with the wild-type strain; and the motility, chemotaxis, and adhesion of *tonB* have weakened by 18.17% (Figures 2A,E), 39.99% (Figure 2B), and 59.34% (Figures 2C,F), respectively, at corresponding periods. Meanwhile, the *tonB*-RNAi strain of *P. plecoglossicida* showed an extremely significant difference (*p* < 0.001) in biofilm formation as compared with the wild-type strain. When the expression of *tonB* was inhibited, the biofilm formation ability of *P. plecoglossicida* decreased at 38.35% (Figure 2D).

### Effects of *tonB* Gene Silencing on the Virulence of *Pseudomonas plecoglossicida*

**FIGURE 3:**
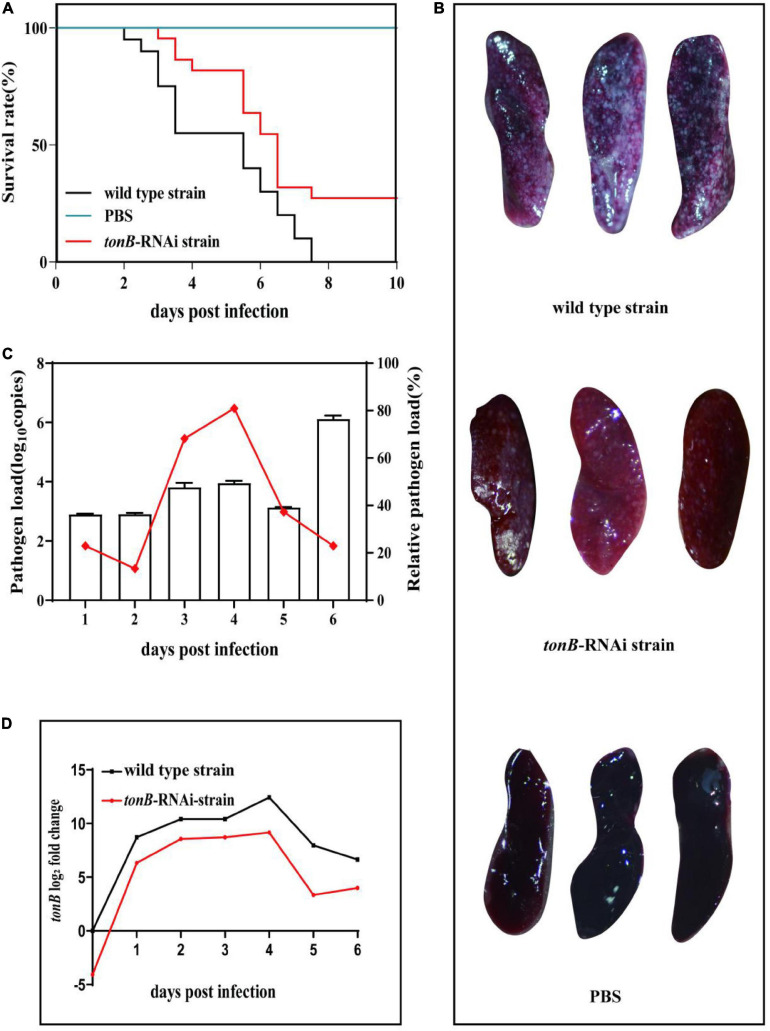
Pathogenicity of two strains of *Pseudomonas plecoglossicida* to *Epinephelus coioides*. **(A)** Survival rate of *E. coioides* infected with wild-type strain or *tonB*-RNAi strain. **(B)** Symptoms of *E. coioides* spleen after infection of wild-type strain or *tonB*-RNAi strain. **(C)** Pathogen load of *tonB*-RNAi strain of *P. plecoglossicida* in the spleens of *E. coioides* during infection. The bar graph represents the pathogen load and is represented by the copy number of *gyrB* gene; the line graph represents the relative pathogen load, which is represented by (copy number of *gyrB* gene of *tonB*-RNAi strain/copy number of *gyrB* gene of wild-type strain). **(D)** mRNA level of *tonB* gene of *P. plecoglossicida* in *E. coioides* spleen during infection.

Infection of the wild-type strain of *P. plecoglossicida* caused the death of *E. coioides*. The first death was recorded at 2 dpi, and the mortality reached 100% at 7.5 dpi. Infection of same dose of the *tonB*-RNAi strain of *P. plecoglossicida* resulted in 1 day delay in the time of first death and 20% decrease in cumulative mortality. No death of *E. coioides* injected with PBS was recorded (Figure 3A).

The surface of *E. coioides* spleens injected with the wild-type strain of *P. plecoglossicida* was covered with numerous white nodules, while much fewer white nodules were found on the surface of counterpart spleens infected with the *tonB*-RNAi strain, and no white nodule was found on the surface of counterpart spleens injected with PBS (Figure 3B).

The pathogen loads of the *tonB*-RNAi strain of *P. plecoglossicida* were always lower than those of the wild-type strain during the whole infection. The relative pathogen load (pathogen load of the *tonB*-RNAi strain/pathogen load of the wild-type strain) of *P. plecoglossicida* peaked at 4 dpi. Although the *tonB*-RNAi strain had a higher pathogen load at 6 dpi than on other times, the relative pathogen load at 6 dpi was lower than that on other times except that at 2 dpi (Figure 3C). The mRNA level of *tonB* gene of *P. plecoglossicida* in the spleen of *E. coioides* was always higher than that *in vitro*, and the highest values were recorded at 4 dpi. Simultaneously, the expression levels of *tonB* in the *tonB*-RNAi strain were always lower than those in the wild-type strain (Figure 3D).

### The Effects of *tonB* Gene on the Immune Response of *Epinephelus coioides* to *Pseudomonas plecoglossicida* Infection

#### Quality Control of RNA-Seq Data

The *E. coioides*’s spleens were subjected to RNA-seq after being infected with the *tonB*-RNAi strain or wild-type strain of *P. plecoglossicida*. The A/T/G/C base content distribution was balanced, and N% met the normative range (Supplementary Figure 2). The main criterion of evaluating the quality of reads was Q20, which fulfilled the requirement of sequencing data of each sample (Q20 > 98%) (Supplementary Figure 3). The base error rate of the sequencing data was <0.1%. Pearson’s correlation coefficients (*r*) showed that three biological replicate samples were closely correlated (*r* > 0.9) (Supplementary Figure 4). The quality of sequence data satisfied the requirements for the subsequent data process and analysis steps.

#### Analysis of Differentially Expressed mRNAs

DESeq2 was used for the analysis of significantly differentially expressed transcripts of *E. coioides*. The criteria of the statistically significant of mRNA expression level changes in the transcriptome data were FDR < 0.05 and | log2FC| ≥ 1. GO and KEGG pathway enrichment analyses were performed for DEMs.

**FIGURE 4:**
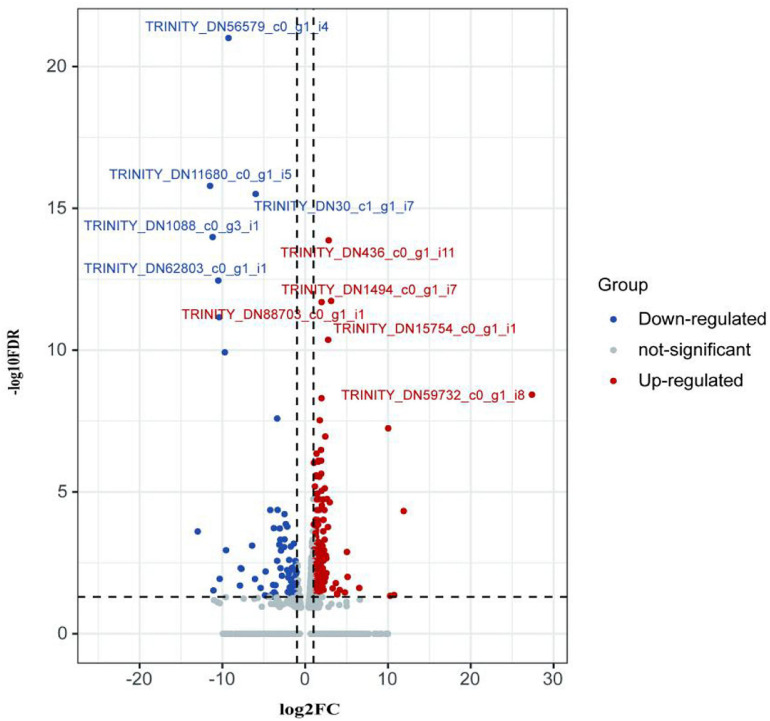
Volcano plot obtained from the DESeq2 analysis of *Epinephelus coioides*’s spleen RNA pools (3 dpi).

**FIGURE 5:**
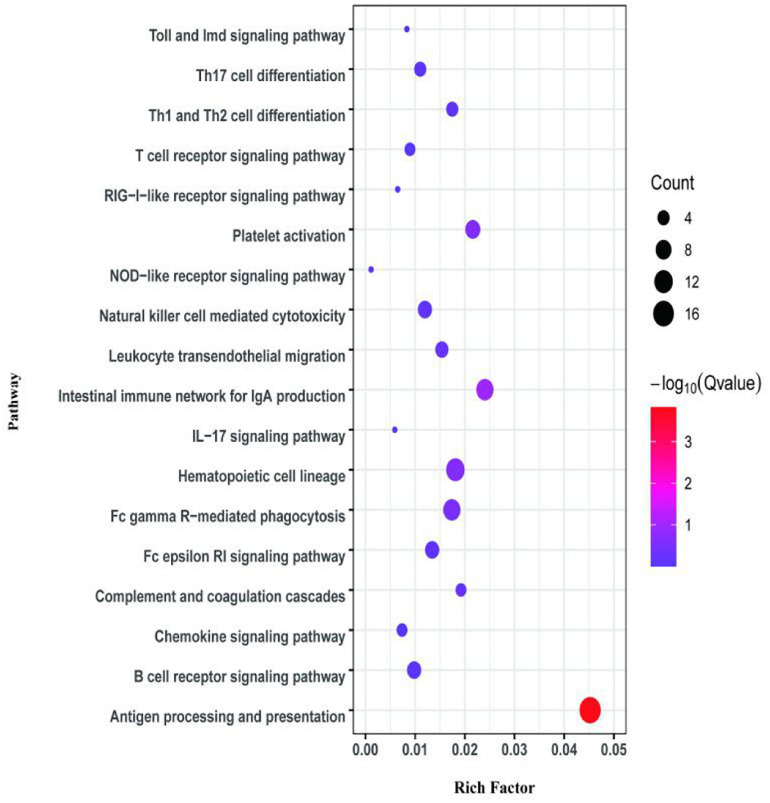
Kyoto Encyclopedia of Genes and Genomes (KEGG) pathway enrichment analysis for differentially expressed mRNAs (DEMs) of transcriptome at 3 dpi.

The first analyzed transcriptome data from the *E. coioides*’s spleens were sampled at 3 dpi. There exist 375 DEMs between the spleen infected with *tonB*-RNAi strains and wild-type strains of *P. plecoglossicida*, which included 291 upregulated mRNAs and 84 downregulated mRNAs (Figure 4). Further KEGG analysis showed that 20 KEGG pathways involved in immune response were enriched (Figure 5). Of the total DEMs, 26.67% were enriched in immune system pathways, including Toll and Imd signaling pathway, Intestinal immune network for IgA production, B-cell receptor signaling pathway, and antigen processing and presentation. The antigen processing and presentation pathway (KO ID: ko04612) was significantly enriched based on the *p*-value of Fisher’s exact test.

**FIGURE 6:**
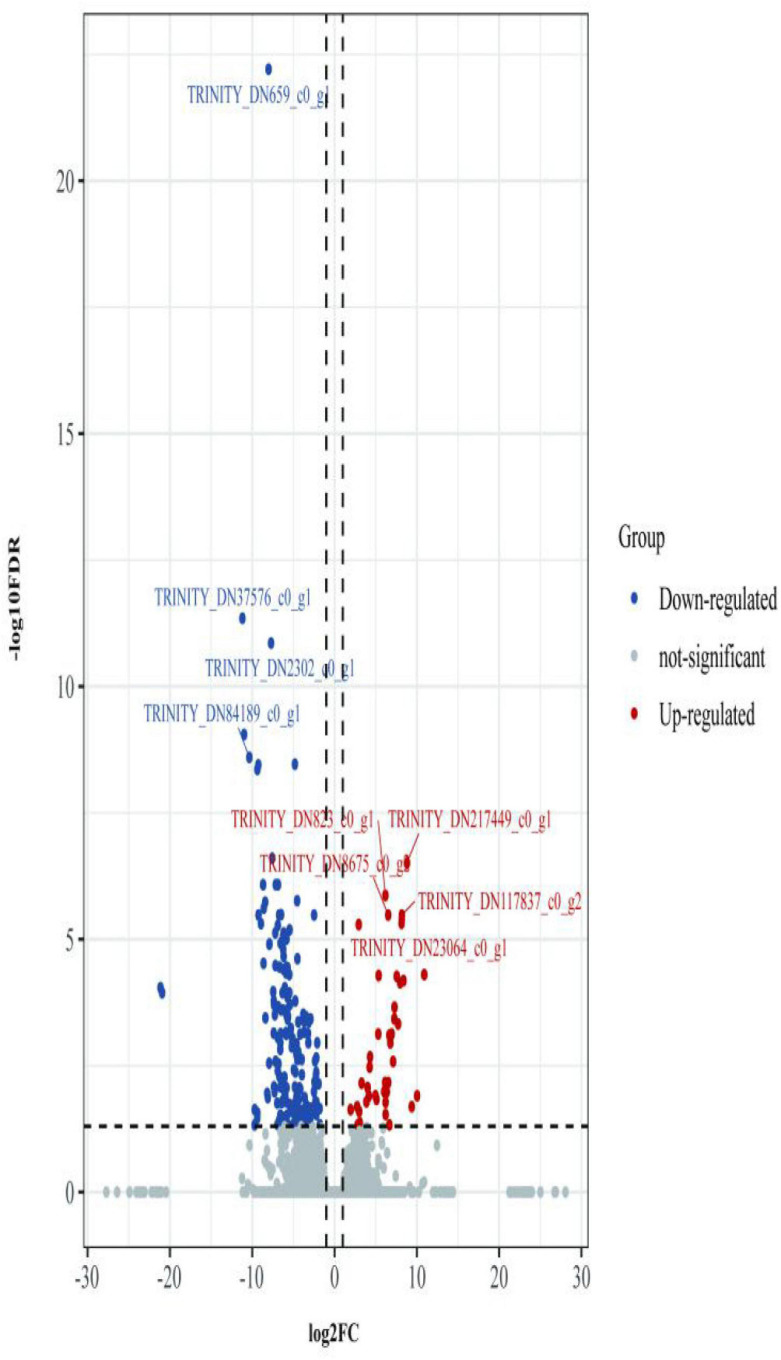
Volcano plot obtained from the DESeq2 analysis of *Epinephelus coioides*’s spleen RNA pools (5 dpi).

**FIGURE 7:**
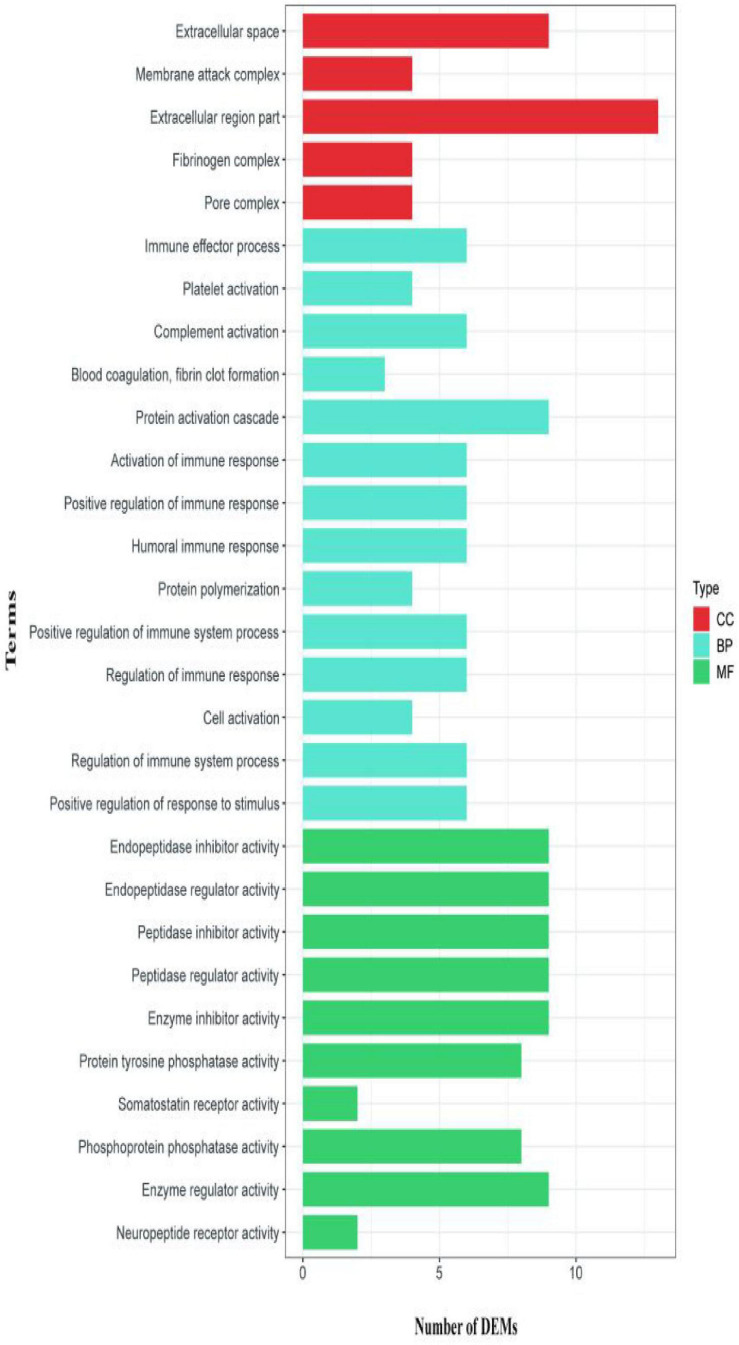
Gene Ontology (GO) enrichment analysis for differentially expressed mRNAs (DEMs) of transcriptome at 5 dpi.

**FIGURE 8:**
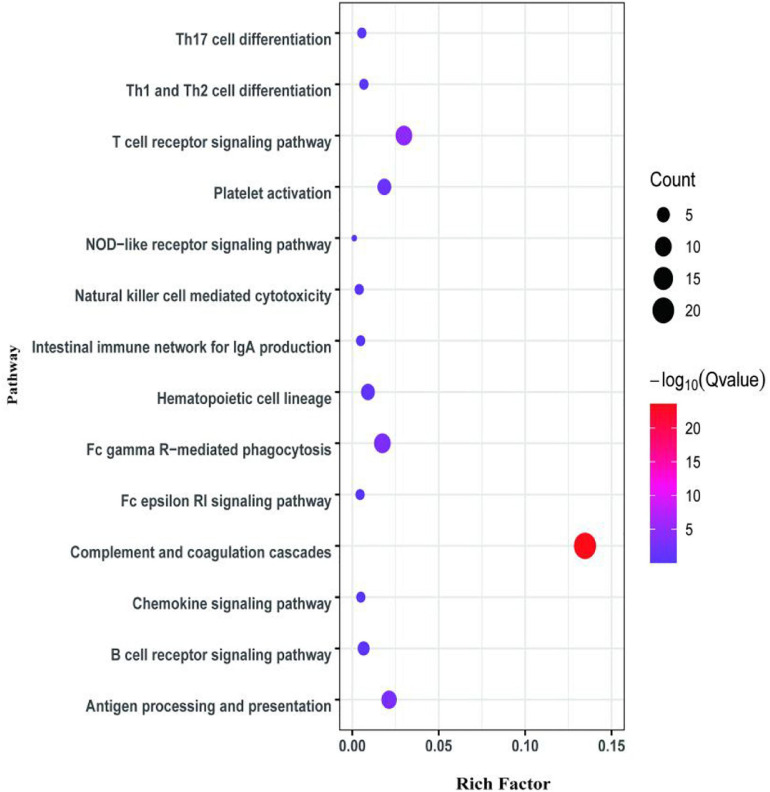
Kyoto Encyclopedia of Genes and Genomes (KEGG) pathway enrichment analysis for differentially expressed mRNAs (DEMs) of the second transcriptome (5 dpi).

The second analyzed transcriptome data from the *E. coioides*’s spleens were sampled at 5 dpi. A total of 218 mRNAs collected from the spleen infected with the *tonB*-RNAi strain of *P. plecoglossicida* were identified as significant differences in expression compared with the spleen infected with the wild-type strain, which included 43 upregulated mRNAs and 175 downregulated mRNAs (Figure 6). According to the GO annotation conventions, DEMs fall into three categories: biological processes, cellular components, and molecular functions. A total of 117 GO terms were enriched, including 29 significantly enriched GO terms. The 29 notably enriched GO terms of the three categories were selected for statistical analysis. The GO analysis results showed that *tonB* gene had a great influence on the immune system, because more than half of the immune GO terms were enriched in biological processes (Figure 7). Of the DEMs, 19.3% were enriched in immune GO terms. According to the KEGG database, 145 KEGG pathways were enriched, including 14 immune-related KEGG pathways. Of the total DEMs, 35.78% were enriched in immune system pathways (Figure 8). Complement and coagulation cascade pathway (ko04610), which included the greatest number of DEMs, was significantly enriched according to the *p*-value of Fisher’s exact test.

**FIGURE 9:**
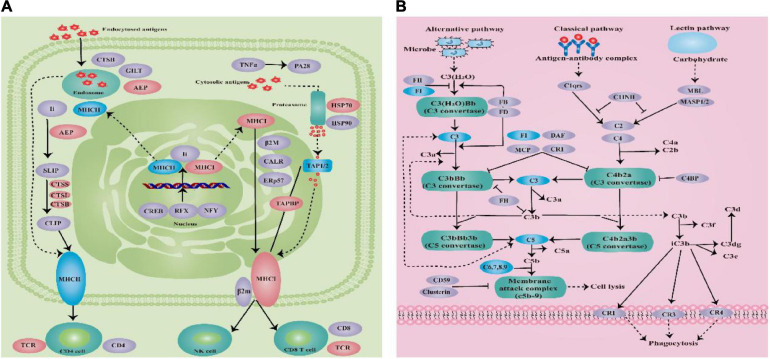
**(A)** Schematic diagram of antigen processing and presentation pathway. **(B)** Schematic diagram of complement and coagulation cascades pathway (blue indicates downregulation, red indicates upregulation, and purple indicates no significant change).

The most significant enrichment pathways were complement and coagulation cascades pathway and antigen processing and presentation pathway according to the KEGG enrichment analysis of the transcriptome data that from the *E. coioides*’s spleens were sampled at 3 and 5 dpi, respectively. Antigen processing and presentation pathway enriched 17 DEMs significantly, which including six downregulated DEMs (such as MHCII and TAP1/2) and 11 upregulated DEMs (such as MHCI, AEP, CTSB/L/S, TCR, TAPBP, and HSP70) (Figure 9A). Complement and coagulation cascades pathway enriched 21 downregulated DEMs significantly (such as FI, C3, C5, C6, C7, C8, and C9) (Figure 9B).

## Discussion

RNA interference has been used for many fields to explore the functions of genes (Belles, 2010; Cooper et al., 2021). In this study, four shRNAs exhibited different silence efficiencies to *tonB* gene, which were consistent with the previous RNAi results (Liu et al., 2020; Xin et al., 2020). The best silence efficiency of four shRNAs to *tonB* gene was 94%, which was higher than that of *pvdE* gene (Xin et al., 2020) but lower than that of *impB* gene (Liu et al., 2020). The stability of gene silencing is crucial to the study of gene function. In the present study, *tonB* gene in the *tonB*-RNAi strain of *P. plecoglossicida* was persistently silenced during the infection process, and the relative expressions of *tonB* gene in the *tonB*-RNAi strain were always lower than those of the wild-type strain. These results indicated that RNAi of *tonB* gene was reliable and laid the foundation for subsequent research.

The TonB protein, encoded by *tonB* gene, has amalgamated to form the TonB system with ExbB and ExbD proteins (Liao et al., 2015). Abdelhamed et al. (2017) investigated the virulence of *tonB* gene of *Edwardsiella ictaluri*, and their findings show that the *E. ictaluri* mutant defective in *tonB* has a 2.16-fold reduction in virulence as compared with the wild-type of *E. ictaluri*. Wang et al. (2008) found that the LD_50_ value of three *tonB* mutant strains of *V. alginolyticus* was increased by 11-fold, 14-fold, and 25-fold, respectively. RNAi of *tonB* gene caused a 20% increase in the survival rate of *E. coioides* to *P. plecoglossicida* infection and had no significant effect on the growth rate of *P. plecoglossicida*, which suggested that the decline in mortality of *E. coioides* was due to the decrease of *P. plecoglossicida* virulence, not due to the decrease of bacterial growth rate. These results suggested that *tonB* gene contributed to the pathogenicity of *P. plecoglossicida*. These results agreed with previous studies of *tonB* gene contribution to the virulence in other bacterial strains. The different influence degrees of *tonB* gene on the pathogenic of different bacteria might be due to the bacterial species, the host species, and the conditions of infection. The results of pathogen load and symptoms agreed with the mortality result. Several genes have been verified to associate with the virulence of *P. plecoglossicida* (Luo et al., 2019). The silencing of some of these genes in *P. plecoglossicida* resulted in lower mortality in experimental fish infected with the mutants (Tang et al., 2019; Wang et al., 2019), and silencing of other virulence genes did not result in the death of *E. coioides* infected with the mutants of *P. plecoglossicida* (Liu et al., 2020; Xin et al., 2020).

RNAi of *tonB* gene attenuated bacterial motility, chemotaxis, adhesion, and biofilm formation of *P. plecoglossicida*. Bacterial motility (Luo et al., 2016), chemotaxis (Zhang et al., 2020), adhesion (Guo et al., 2018), and biofilm formation (Zhang et al., 2019) have been demonstrated to be related to the pathogenic of pathogen. Some studies showed that *tonB* mutant had great influences on motility, adhesion, and *in vivo* virulence due to its ability of helping the formation of type IV pili (Huang et al., 2004; Duong-Nu et al., 2016).

Nowadays, transcriptome analysis is an important way to reveal the mechanisms of host immune response to infection (Wu and Chen, 2016; Song et al., 2017; Li et al., 2021). In this study, compared with the transcriptome data from *E. coioides* infected with the wild strain of *P. plecoglossicida*, significant changes of transcriptome were observed in the spleen of *E. coioides* infected with the *tonB*-RNAi strain. The GO analysis results showed that the *tonB*-RNAi strain had a great influence on the immune system, and more than half of the immune pathways were enriched in biological processes. The KEGG pathway analysis results showed that the most significant pathways for enrichment were complement and coagulation cascades pathway and antigen processing and presentation pathway.

Complement and coagulation cascades pathway is an immune defense mechanism of the host, and the complement cascades are closely associated with coagulation cascades to jointly achieve effective protection of the host (Lupu et al., 2014; Wiegner et al., 2016). It is widely known that the activation and generation of C3a and C5a have serious proinflammatory effects (Pontrelli et al., 2020). Compared with the transcriptome data from *E. coioides* infected with the wild-type strain of *P. plecoglossicida*, all of the DEMs in transcriptome data from *E. coioides* infected with the *tonB*-RNAi strain were significantly downregulated in the complement and coagulation cascades pathway, which indicated that the inflammatory reaction significantly declined in *E. coioides* infected with the *tonB*-RNAi strain of *P. plecoglossicida*.

The antigen processing and presentation pathway plays a crucial role in immunological process, which is presented by major histocompatibility complexes (MHCs) (Park and Jae-Hwan, 2011; Wieczorek et al., 2017). MHCI relies on proteasomal proteolysis to present foreign peptides that come from degradation of endocellular microbial pathogens; MHCII depends upon lysosomal degradation to accomplish the presentation and processing of extracellular antigens (Wilson, 2017). Therefore, the determining factor of the successful pathogen elimination obviously depends on the recognition of MHCs in the immune system (Unanue, 2002). Compared with the transcriptome data from *E. coioides* infected with the wild-type strain of *P. plecoglossicida*, most of the DEMs in transcriptome data from *E. coioides* infected with the *tonB*-RNAi strain related with MHCI and MHCII were significantly upregulated in the antigen processing and presentation pathway. These results indicated that *E. coioides*’s immune system could more efficiently identify the *tonB*-RNAi strain of *P. plecoglossicida*, which might cause the immune system to remove the mutant strain more efficiently.

## Conclusion

In conclusion, *tonB* is a virulence gene of *P. plecoglossicida*; *tonB* gene is involved in the regulation of bacterial motility, chemotaxis, adhesion, and biofilm formation of *P. plecoglossicida*; RNAi of *tonB* gene significantly affected the immune response of *E. coioides* to *P. plecoglossicida* infection; complement and coagulation cascades pathway and antigen processing and presentation pathway are the most affected immune pathways.

## Data Availability Statement

The datasets presented in this study can be found in online repositories. The names of the repository/repositories and accession number(s) can be found in the article/Supplementary Material.

## Ethics Statement

The animal study was reviewed and approved by the Animal Ethics Committee of Jimei University (Acceptance No. JMULAC201159).

## Author Contributions

All authors contributed to the article. QY, LL, ZZ, and XW conceived the experiments. LFH, LL, and LZ conducted the experiments. All authors assisted in the collection and interpretation of data. LFH, ZZ, and QY wrote the manuscript.

## Conflict of Interest

JZ and QY were employed by company Fujian Tianma Technology Company Limited. The remaining authors declare that the research was conducted in the absence of any commercial or financial relationships that could be construed as a potential conflict of interest.

## Publisher’s Note

All claims expressed in this article are solely those of the authors and do not necessarily represent those of their affiliated organizations, or those of the publisher, the editors and the reviewers. Any product that may be evaluated in this article, or claim that may be made by its manufacturer, is not guaranteed or endorsed by the publisher.

## Footnotes

1
http://rnaidesigner.thermofisher.com/rnaiexpress/setOption.do?designOption=shrna&pid=708587103220684543

2
https://github.com/jstjohn/SeqPrep

3
https://github.com/najoshi/sickle

4
https://www.blast2go.com/

## Supplementary Material

Supplementary Figure 1 Verification of transcriptome data with qRT-PCR.

Supplementary Figure 2 Bases content along raw reads.

Supplementary Figure 3 Mean errors distribution along raw reads.

Supplementary Figure 4 Correlation of transcriptional data.

Supplementary Table 1 The sequences of four shRNAs for *tonB* gene.

Supplementary Table 2 The sequence of Primers for PCR and qRT-PCR.
