# Supplementary material for: The Effect of tonB Gene on the Virulence of Pseudomonas plecoglossicida and the Immune Response of Epinephelus coioides
Source: Front Microbiol. 2021 Aug 16;12:720967. doi: 10.3389/fmicb.2021.720967 (PMC8415555; doi:10.3389/fmicb.2021.720967)
Supplement: Supplementary Figure 1 — Verification of transcriptome data with qRT-PCR. [file Data_Sheet_1.docx]

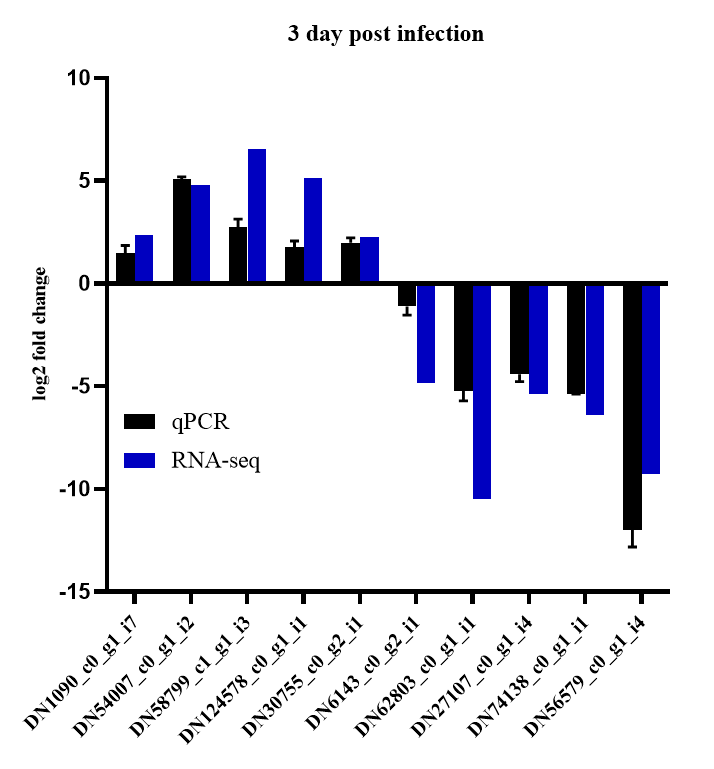


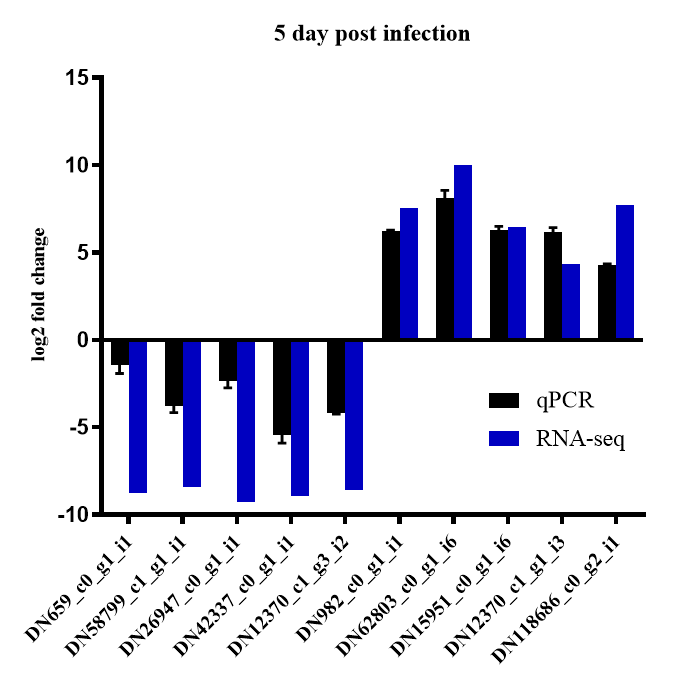


**Supplementary Fig. 1: Verification of transcriptome data with qRT-PCR**. The RNA-seq data were shown on blue columns and the qRT-PCR data were shown on black column
