# Supplementary material for: The Effect of tonB Gene on the Virulence of Pseudomonas plecoglossicida and the Immune Response of Epinephelus coioides
Source: Front Microbiol. 2021 Aug 16;12:720967. doi: 10.3389/fmicb.2021.720967 (PMC8415555; doi:10.3389/fmicb.2021.720967)
Supplement: Supplementary Figure 2 — Bases content along raw reads. [file Data_Sheet_2.doc]

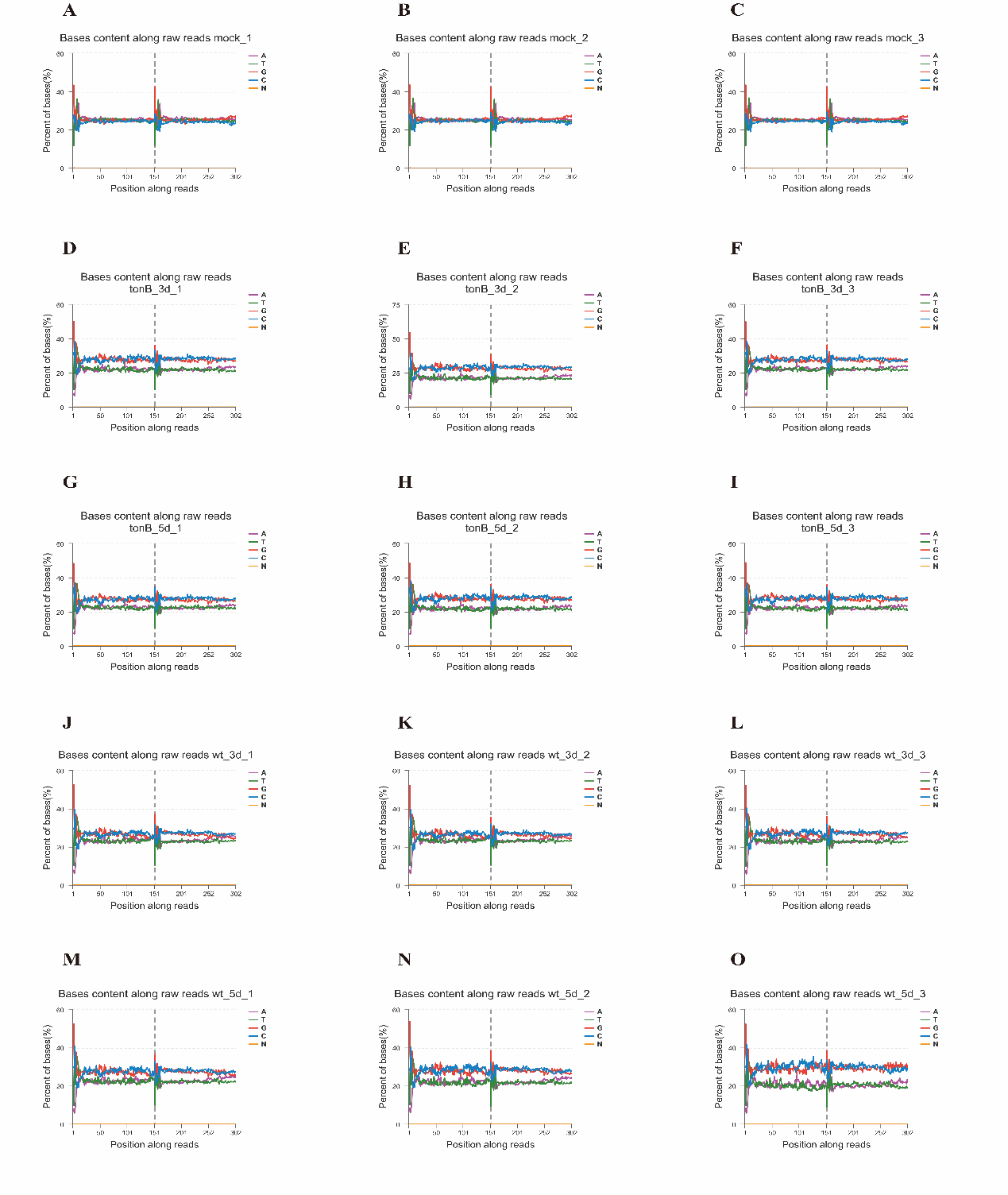


**Supplementary Fig. 2:** **Bases content along raw reads** (shows whether there is separation of AT and GC base). The abscissa is the bases of reads, and the ordinate is all reads at the sequencing position. Each base with different color: A is purple; T is green; G is red; C is blue; N is orange. (A), (B), (C) are PBS injected group; (D), (E), (F) are *tonB*-RNAi strain infected group(3d) ; (G), (H), (I) are *tonB*-RNAi strain infected group(5d); (J), (K), (L) are wild type strain infected group(3d); (M), (N), (O) are wild type strain infected group(5d)
