# Supplementary material for: The Effect of tonB Gene on the Virulence of Pseudomonas plecoglossicida and the Immune Response of Epinephelus coioides
Source: Front Microbiol. 2021 Aug 16;12:720967. doi: 10.3389/fmicb.2021.720967 (PMC8415555; doi:10.3389/fmicb.2021.720967)
Supplement: Supplementary Figure 3 — Mean errors distribution along raw reads. [file Data_Sheet_3.doc]

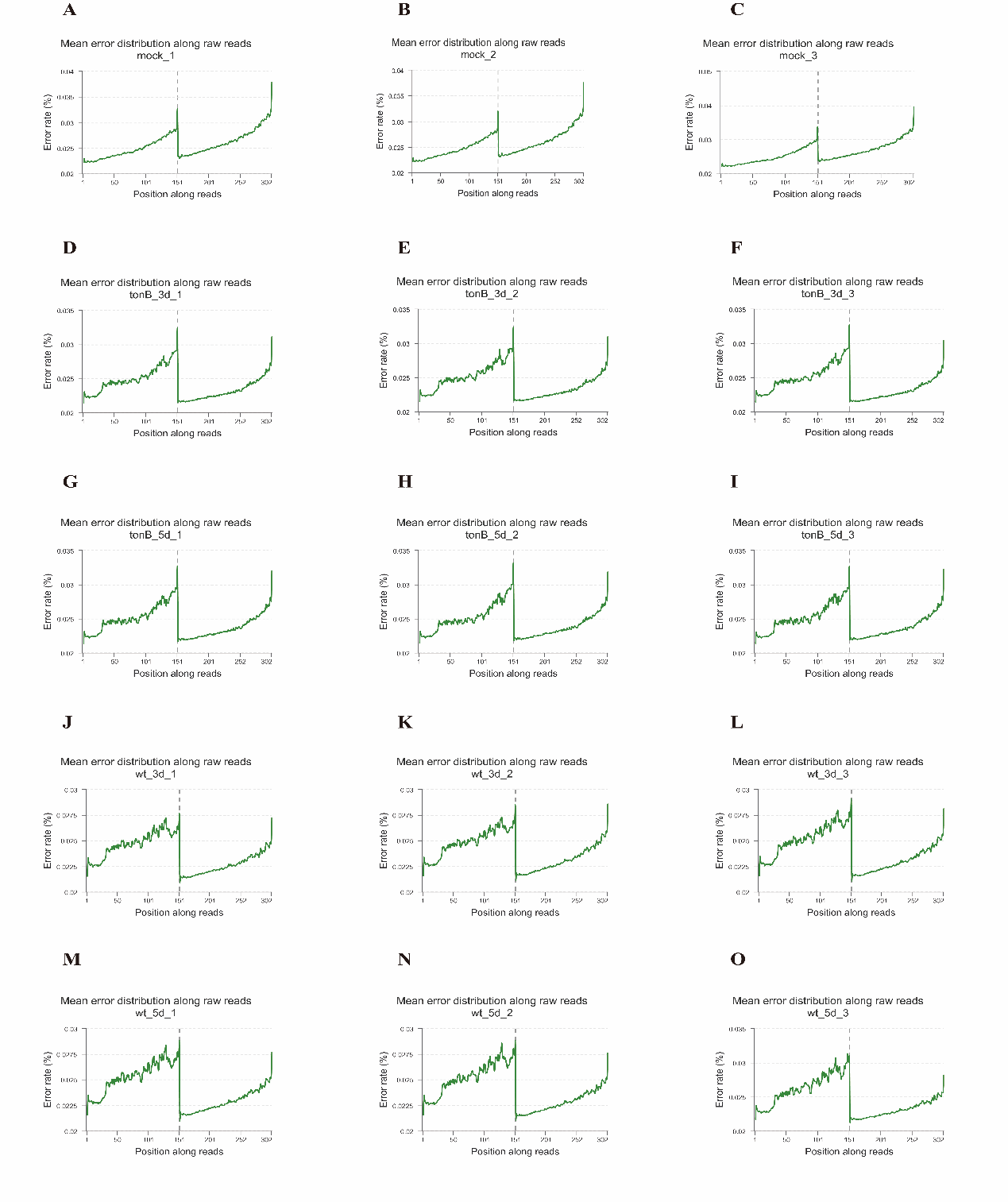


**Supplementary Fig. 3: Mean errors distribution along raw reads.** The abscissa is the sequence of bases from 5’ to 3’ in reads and the ordinate is the average error rate (%) of all reads at this site. The green line in the graph corresponds to the average value of the base error rate, which reflects the distribution of base error rate in the sequencing reads. (A), (B), (C) are PBS injected group; (D), (E), (F) are *tonB*-RNAi strain infected group(3d) ; (G), (H), (I) are *tonB*-RNAi strain infected group(5d); (J), (K), (L) are wild type strain infected group(3d); (M), (N), (O) are wild type strain infected group(5d)
