# Supplementary material for: The Effect of tonB Gene on the Virulence of Pseudomonas plecoglossicida and the Immune Response of Epinephelus coioides
Source: Front Microbiol. 2021 Aug 16;12:720967. doi: 10.3389/fmicb.2021.720967 (PMC8415555; doi:10.3389/fmicb.2021.720967)
Supplement: Supplementary Figure 4 — Correlation of transcriptional data. [file Data_Sheet_4.doc]

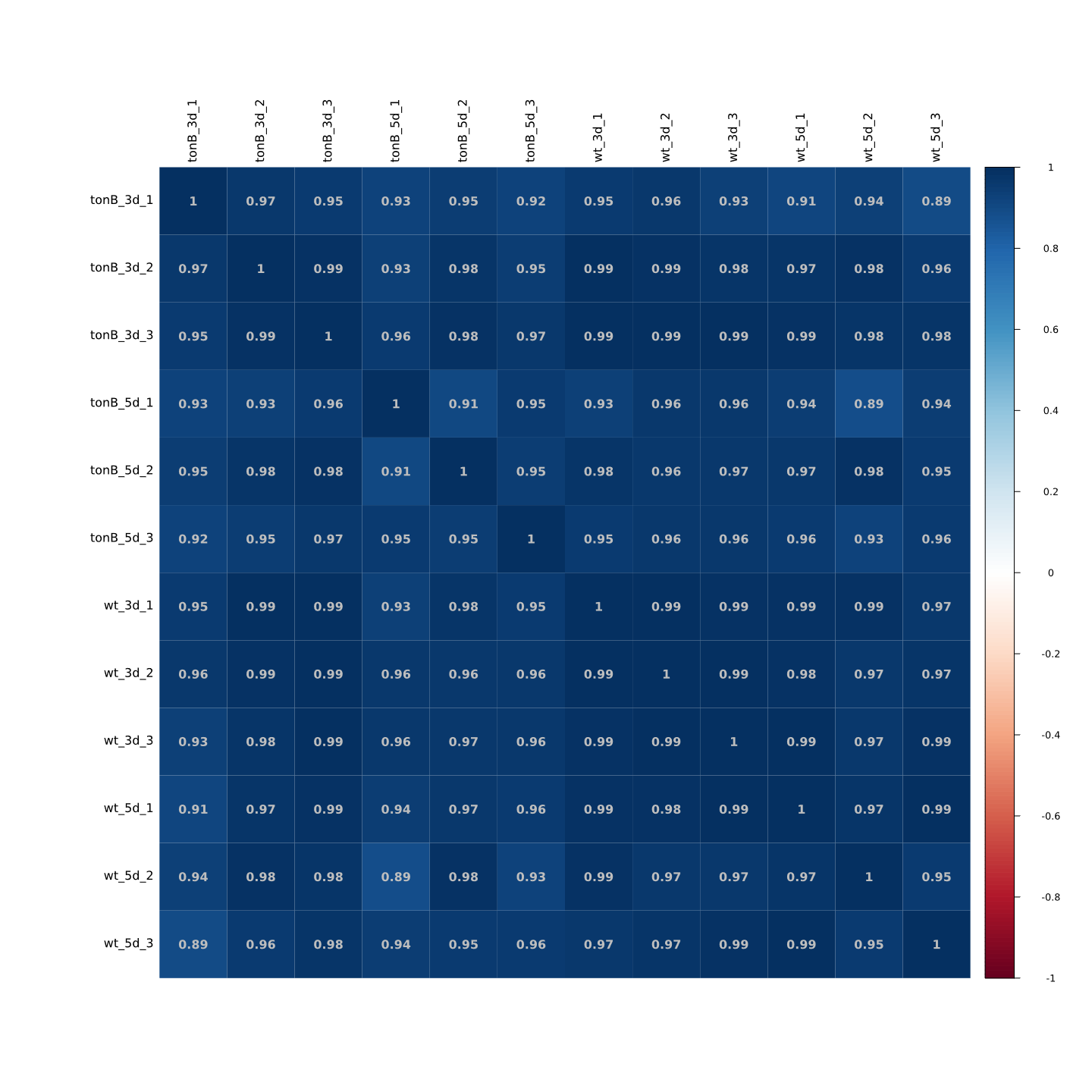


**Supplementary Fig. 4: Correlation of transcriptional data.** The value in the square indicates the correlation coefficient between the corresponding two samples (based on the Pearson Correlation Coefficient). The larger the correlation coefficient, the darker the color, the greater the correlation between the two samples.
