# Supplementary material for: The Effect of tonB Gene on the Virulence of Pseudomonas plecoglossicida and the Immune Response of Epinephelus coioides
Source: Front Microbiol. 2021 Aug 16;12:720967. doi: 10.3389/fmicb.2021.720967 (PMC8415555; doi:10.3389/fmicb.2021.720967)
Supplement: Supplementary Table 1 — The sequences of four shRNAs for tonB gene. [file Table_1.doc]

**Supplementary Table 1**: The sequences of four shRNAs for *tonB* gene

| **Name** | **Base sequence** |
| --- | --- |
| ***tonB*-RNAi-419** | F: 5’-TGCATCGCTTCGAACAGCAAAGTTCAAGAGACTTTGCTGTTCGAAGCGATGCTTTTTTT-3’  R: 5’-GTACAAAAAAAGCATCGCTTCGAACAGCAAAGTCTCTTGAACTTTGCTGTTCGAAGCGATGCATGCA-3’ |
| ***tonB*-RNAi-424** | F: 5’-TGCTTCGAACAGCAAAGCCTTGTTCAAGAGACAAGGCTTTGCTGTTCGAAGCTTTTTTT-3’  R: 5’-GTACAAAAAAAGCTTCGAACAGCAAAGCCTTGTCTCTTGAACAAGGCTTTGCTGTTCGAAGCATGCA-3’ |
| ***tonB*-RNAi-663** | F: 5’-TGCCACCTGAGTTGCTGAACAATTCAAGAGATTGTTCAGCAACTCAGGTGGCTTTTTTT-3’  R: 5’-GTACAAAAAAAGCCACCTGAGTTGCTGAACAATCTCTTGAATTGTTCAGCAACTCAGGTGGCATGCA-3’ |
| ***tonB*-RNAi-675** | F: 5’-TGCTGAACAATGGCACGATCGATTCAAGAGATCGATCGTGCCATTGTTCAGCTTTTTTT-3’  R: 5’-GTACAAAAAAAGCTGAACAATGGCACGATCGATCTCTTGAATCGATCGTGCCATTGTTCAGCATGCA-3’ |
