# Supplementary material for: The Effect of tonB Gene on the Virulence of Pseudomonas plecoglossicida and the Immune Response of Epinephelus coioides
Source: Front Microbiol. 2021 Aug 16;12:720967. doi: 10.3389/fmicb.2021.720967 (PMC8415555; doi:10.3389/fmicb.2021.720967)
Supplement: Supplementary Table 2 — The sequence of Primers for PCR and qRT-PCR. [file Table_2.doc]

| **Supplementary Table 2**: The sequence of Primers for PCR and qRT-PCR | |
| --- | --- |
| **gene name** | Base sequence (From 5' to 3') |
| ***gyrB*** | F: 5'-TGCTGAAGGACGAGCGTTCG-3' |
|  | R: 5'-ATCATCTTGCCGACAACAGC-3' |
| ***tonB*** | F: 5'-GTGGACACGCCGCCAAGCAA-3' |
|  | R: 5'-GCCAGGTGACGCAGCAAATCG-3' |
| ***16S rDNA*** | F: 5'-TCAGTATCAGTCCAGGTGGTCGC-3' |
|  | R: 5'-CGTTACCGACAGAATAAGCACCG-3' |
| ***pcM130 / tac*** | F: 5'-CTTCCTGGTTGGCTTGGTTTC-3' |
|  | R: 5'-GGTGTTCCTTCTTCACTGTCCCT-3' |
| ***β-actin*** | F: 5'-GGCTACTCCTTCACCACCACA-3' |
|  | R: 5'-GGGCAACGGAACCTCTCAT-3' |
| ***DN58799_c1_g1_i3*** | F: 5'-GGTGATGACAGCCTGTTGTTT-3' |
|  | R: 5'-TGGGACGATGAGACTGGAATA-3' |
| ***DN124578_c0_g1_i1*** | F: 5'-ATCTACTCCCTCACCTCTGC-3' |
|  | R: 5'-ACTCTGGCTGGTCACATCTC-3 |
| ***DN54007_c0_g1_i2*** | F: 5'-GTTATTATCACAAAGCCAAAGG-3' |
|  | R: 5'-CTCATCAGAGGAGGAGGTCA-3' |
| ***DN1090_c0_g1_i7*** | F: 5'-CTACACTCGTCCCACTCC-3' |
|  | R: 5'-CACTGGCAGCAAGAAAGC-3' |
| ***DN30755_c0_g2_i1*** | F: 5'-CAAAGGACTCTATGGACAGC-3' |
|  | R: 5'-AAAAGCATAAAGAAGGCAGT-3' |
| ***DN74138_c0_g1_i1*** | F: 5'-AACAGGCTTTCATCACCAA-3' |
|  | R: 5'-AGCTCCTCCCATAGTCCAA-3' |
| ***DN62803_c0_g1_i1*** | F: 5'-TTTCATTGCCAGCACCATC-3' |
|  | R: 5'-TGTCGCACCCATCACCTCT-3' |
| ***DN56579_c0_g1_i4*** | F: 5'-CAAAGACGACCAGTCCAAC-3' |
|  | R: 5'-ACCTACGTGTCACAAACCC-3' |
| ***DN27107_c0_g1_i4*** | F: 5'-TATGTGCAACCAGGAGGAGC-3' |
|  | R: 5'-CAGCGACCAGCAGGATGTAG-3' |
| ***DN6143_c0_g2_i1*** | F: 5'-TTTGAGGGAAGATGGGTT-3' |
|  | R: 5'-CGGTAGGCTTGGGTGTAT-3' |
| ***DN62803_c0_g1_i6*** | F: 5'-TTTCATTGCCAGCACCATC-3' |
|  | R: 5'-ACCACTGTTCAGCCCTTCA-3' |
| ***DN118686_c0_g2_i1*** | F: 5'-GGATGTGCTTCTGGAGGTCGTA-3' |
|  | R: 5'-AGGTGGAAATCTCACTAAAATG-3' |
| ***DN982_c0_g1_i1*** | F: 5'-CCTGTAAACCCGAACTGATTGT-3' |
|  | R: 5'-TGCGTAGCCGTGTCTATTGATT-3' |
| ***DN15951_c0_g1_i6*** | F: 5'-TTGGTGGTCTCAGTGTTTCC-3' |
|  | R: 5'-GTCCTCTGTCCTGCTGGTCT-3' |
| ***DN12370_c1_g1_i3*** | F: 5'-TTGGTGGTCTCAGTGTTTCC-3' |
|  | R: 5'-GTCCTCTGTCCTGCTGGTCT-3' |
| ***DN659_c0_g1_i1*** | F: 5'-TGCAACCGAGTTCATCCT-3' |
|  | R: 5'-TGTGCCAACACTTTCAGC-3' |
| ***DN26947_c0_g1_i1*** | F: 5'-TTACCAGAGTGATTGGGACA-3' |
|  | R: 5'-GTGATGCTTTTAGTGAGGCT-3' |
| ***DN42337_c0_g1_i1*** | F: 5'-CATTAGGTTCCACATCCCAGTA-3' |
|  | R: 5'-ATTTGTCAACCATCAGCCTCT-3' |
| ***DN12370_c1_g3_i2*** | F: 5'-AGGAGACAATCAGCCATCAGA-3' |
|  | R: 5'-ACAGGGTAAAGGAGCAGCATC-3' |
| ***DN58799_c1_g1_i1*** | F: 5'-TATTCCAGTCTCATCGTCCCA-3' |
|  | R: 5'-GCAGAACTTCAAAGCCAACAT-3' |
